# Real-world glycemic control, exploratory cardiorenal indicators, and safety of polyethylene glycol loxenatide versus semaglutide in type 2 diabetes patients: a Chinese two-center retrospective cohort study

**DOI:** 10.3389/fendo.2026.1756581

**Published:** 2026-03-24

**Authors:** Zelin Yu, Duoyi Fu, Jing Xu, Chujia Lin, Guoshu Yin, De Cai, Hongyan Jiang, Kai Ling, Jianqin Chen, Yeye Zhuo

**Affiliations:** 1 Department of Clinical Pharmacy, The Second Affiliated Hospital of Shantou University Medical College, Shantou, China; 2 Department of Biology, School of Science, Shantou University, Shantou, China; 3 Department of Clinical Pharmacy, The First Affiliated Hospital of Shantou University Medical College, Shantou, China; 4 Department of Endocrinology, The First Affiliated Hospital of Shantou University Medical College, Shantou, China; 5 Department of Pharmacology, Shantou University Medical College, Shantou, China; 6 Department of Geriatric Medicine, The First Affiliated Hospital of Shantou University Medical College, Shantou, China

**Keywords:** polyethylene glycol loxenatide, s.c. semaglutide, type 2 diabetes mellitus, HbA1c, body weight, lipid profiles, estimated glomerular filtration rate, adverse drug events

## Abstract

**Objective:**

To compare the real-world efficacy and safety of high dose once-weekly glucagon-like peptide-1 receptor agonists polyethylene glycol loxenatide (PEG-Loxe) and subcutaneous (s.c.) semaglutide in patients with suboptimally controlled type 2 diabetes mellitus (T2DM).

**Methods:**

We conducted a retrospective cohort China two-center study. Patients newly initiated on once-weekly PEG-Loxe 200 μg or s.c. semaglutide 1.0 mg between January 2021 and October 2022 were matched 1:1 using propensity score matching. The primary endpoint was change from baseline in glycated hemoglobin (HbA1c) at 24 months. Secondary endpoints included changes in HbA1c, body weight (BW), body mass index (BMI), low-density lipoprotein cholesterol (LDL-C), systolic blood pressure (SBP), and estimated glomerular filtration rate (eGFR) from baseline to 3, 6, 12, and 24 months. Records of adverse drug reactions were collected and extracted from the institutional medical record system.

**Results:**

A total of 510 matched patient pairs demonstrated balanced baseline characteristics. No statistically significant between-group difference was observed in HbA1c changes from baseline across all follow-up assessments (*p* > 0.05). At month 24, HbA1c was reduced by least-squares mean 2.02% with PEG-Loxe and 2.23% with s.c. semaglutide (estimated treatment difference [ETD] -0.21%, 95% CI: -0.52, -0.10, *p* = 0.260). Semaglutide was consistently and significantly superior to PEG-Loxe in improving BW (ETD at 24 months: -3.55 kg, 95% CI: -5.22, -1.88, *p* < 0.001), BMI (ETD -0.82 kg/m^2^, 95%CI: -1.33, -0.32, *p* = 0.001), LDL-C (ETD -0.51 mmol/L, 95% CI: -0.68, -0.34, *p* < 0.001), SBP (ETD -2.08 mmHg, 95% CI: -9.33, -0.11, *p* < 0.001), eGFR [ETD 0.75 mL/(min·1.73m^2^), 95% CI: -2.53, 4.039, *p* < 0.001]. Gastrointestinal symptoms were the most common safety concern, occurring more frequently in the semaglutide group during the first 3 months (*p* < 0.05).

**Conclusions:**

In real-world clinical practice, at the same high dose, PEG-Loxe provided equivalent glycemic control to s.c. semaglutide with a potential lower incidence of gastrointestinal side effects. Conversely, semaglutide offered more substantial improvement of cardiorenal parameters. These findings highlight the clinical utility of both agents in personalized T2DM management.

## Introduction

Glucagon-like peptide-1 receptor agonists (GLP-1RAs) achieve glycemic control primarily through incretin regulation. Moreover, they may reduce body weight (BW), modulate lipid levels, and confer cardiorenal protection in individuals with type 2 diabetes mellitus (T2DM) (1, 2). Once-weekly formulations are associated with higher treatment satisfaction compared to daily regimens.

Currently, both polyethylene glycol loxenatide (PEG-Loxe) and semaglutide were available weekly GLP-1RAs in Chinese clinical settings, with high prescription rate. PEG-Loxe, developed in China, is derived from exendin-4 with PEG modification (3). PEG modification delays Loxe degradation by dipeptidyl peptidase-4 (DPP-4), reduces renal clearance, increases hydrophilicity, and improves stability and circulating half-life. The approved weekly doses of PEG-Loxe in China are 100 μg and 200 μg. Due to the superior efficacy on glycemic control, the application of PEG-Loxe at 200 μg is more extensive in Chinese clinical practice (4). Semaglutide, sharing 94% amino acid sequence homology with native human GLP-1 (5), is available via subcutaneous (s.c.) (approved doses: 0.25, 0.5 and 1.0 mg) and oral administration. Semaglutide carries structural modifications on its GLP-1 moiety, including the conjugation of a fatty diacid chain and two amino acid substitutions. These alterations extend the half-life through enhanced albumin association and inhibition of DPP-4 degradation, enabling once-weekly administration. Remarkably, the recent published cost-effectiveness modeling suggested that, compared with other approved GLP-1 RA/metformin combination in China, PEG-Loxe might represent an economic treatment option (6).

To date, however, no head-to-head studies have evaluated PEG-Loxe versus s.c. semaglutide on efficacy and safety. In phase 3a/3b trials, PEG-Loxe 200 μg demonstrated significant glycated hemoglobin (HbA1c) reductions of 1.14% (monotherapy) - 1.34% (combination with metformin) after 24 weeks (7, 8), with low hypoglycemia rates (0.6%-1.7%) and manageable gastrointestinal adverse events (17.4%-25%). Meanwhile, HbA1c decreased by 1.55% with 1.0 mg s.c. semaglutide at week 30 (9) and 1.6% at week 56 (10) in randomized controlled trials (RCTs), with a <1% incidence of hypoglycemia and 38%-40% rate of gastrointestinal disorders. (RCTs) often lack generalizability due to strict inclusion criteria and short observation periods (11). Real-world studies serve as a robust approach to redressing this limitation, facilitating the assessment of the real-world efficacy and safety profile of pharmaceuticals across a more extensive and heterogeneous patient cohort (12).

What’s more, compared with western populations, East Asian patients with T2DM, including Chinese, exhibit poorer β-cell function and lower insulin resistance (13). Additionally, the most frequently reported type of adverse events (AEs) in GLP-1RAs-treated patients were gastrointestinal AEs. In SUSTAIN China, after 30 weeks of treatment, gastrointestinal AEs occurred in 44.5% of patients with s.c. semaglutide 1.0 mg (14). This incidence rate was slightly higher than that reported in the SUSTAIN 1 study (38%), which was carried out at 72 sites (10). A high incidence of gastrointestinal discomfort may compromise patients’ medication adherence (15). Long-term comparative data between the two agents in routine clinical practice may aid in the identification of tailored treatment plans.

Different chemical compositions, pharmacokinetic characteristics, and pharmacodynamic activities of various GLP-1RAs, may translate to disparities in their clinical effectiveness and safety outcomes (16, 17). Therefore, we hypothesized that there may be also differences in efficacy and safety profiles between PEG-Loxe and semaglutide in real-world practice, and conducted a retrospective cohort study among Chinese population to explore these potential distinctions in a long follow-up period-2 years. Given the rapidly rising incidence and disease burden of T2DM across China over the past 30 years, we focused not only on their effectiveness on glycemic control and AEs, but also amelioration of exploratory cardiorenal indicators, with the hope of improving the management of T2DM.

## Materials and methods

### Study design

This observational retrospective cohort study was conducted in two Chinese primary care institutions with 1,800 and 1,350 beds respectively. Anonymous data were manually extracted from electronic medical records (EMRs) of patients newly initiated on PEG-Loxe or subcutaneous (s.c.) semaglutide between January 2021 and October 2022. The study adhered to the ethical principles of the Declaration of Helsinki. Ethical approval was secured from the institutional review boards of the First and Second Affiliated Hospitals of Shantou University Medical College (approval no. B-2025–045 and 2025-49). Informed consent was obtained from all individual participants included in the study. Informed consent was waived for retrospective use of de-identified clinical data beyond initial treatment consent.

### Study population

The inclusion criteria included the following:

- Age ≥18 years with confirmed diagnosis of T2DM for ≥ 3 months, based on American Diabetes Association criteria 2020) (18).
- Ongoing antihyperglycemic therapy after diagnosis (oral hypoglycemic agents [OHAs] and/or insulin.
- No previous exposure to any GLP-1RAs prior to study entry.
- Initiation of PEG-Loxe (100 μg for 4 weeks followed by 200 μg maintenance) or s.c. semaglutide (0.25 mg for 4 weeks, escalating every 4 weeks to 1.0 mg maintenance) according to the clinical guidelines.
- Undergoing outpatient follow-up every 3–6 months during the first two years following the initiation of PEG-Loxe or semaglutide therapy.

The exclusion criteria included the following:

- Other types of diabetes.
- History of pancreatitis or pancreatic cancer.
- eGFR < 30 mL/(min·1.73m^2^).
- Child-Pugh grade C/liver failure.
- With at least one missing measurement: either baseline HbA1c or the HbA1c at 3, 6, 12, or 24 months.

### Data collection

Baseline data (3 months before the first prescription date) included participant characteristics, diabetes duration, HbA1c, SBP, diastolic blood pressure (DBP), total cholesterol (TC), triglyceride (TG), high-density lipoprotein cholesterol (HDL-C), LDL-C, glutamic-pyruvic transaminase (ALT), glutamic oxalacetic transaminase (AST), serum creatinine (Scr), eGFR, hemoglobin (HGB), baseline antihyperglycemic agents (OHAs and insulin), lipid-lowering therapy, comorbidities, and complication indices (Charlson Comorbidity Index [CCI], adapted Diabetes Complication Severity Index [aDCSI]).

Follow-up data at 3, 6, 12, and 24 months included HbA1c, BW, BMI, SBP, LDL-C, eGFR, antihyperglycemic regimen, and ADEs recorded in outpatient EMRs over the 2-year period.

Outpatient routine practice involves assessing medication adherence in patients with T2DM using the 5-item Medication Adherence Report Scale (MARS-5) (19) upon initial diagnosis and yearly following the confirmation of the disease. The demand of the use of MARS-5 was granted by the originators. Data on medication adherence assessment were retrieved from EMRs.

### Outcome measures

Primary endpoints was HbA1c change from baseline at 24 months.

The Secondary endpoints included the following:

- HbA1c changes from baseline to 3, 6, 12 months.
- BW, BMI, LDL-C, SBP, eGFR changes, from baseline to 3, 6, 12, 24 months.
- Incidence rates of gastrointestinal AEs and hypoglycemic events during each respective time period.
- Composite endpoints including HbA1c < 7.0%, HbA1c ≤ 6.5%, and HbA1c reduction ≥1%, all without hypoglycemia and BW gain.

Subgroup analyses examined HbA1c changes from baseline at 12 and 24 months, alongside the proportion of patients achieving the HbA1c target (<7%) at these time points. Stratification was performed by age ≥ 60, diabetes duration ≥ 5 years, baseline HbA1c ≥ 8.0%, BMI ≥ 24 kg/m², and baseline insulin use.

### Statistical analysis

A minimum sample size of 196 patients was calculated to achieve 80% statistical power with a type I error rate of α = 0.05, for detecting the prespecified hypothetical reductions in HbA1c of 1.2% in the PEG-Loxe group versus 1.5% in the s.c. semaglutide group at the 24-month assessment.

Missing data were addressed using multiple imputation, with a maximum missing data proportion of 4.63% across baseline and follow-up variables. Continuous data that were normally distributed were presented as mean ± standard deviation (mean ± SD); those that were non-normally distributed were expressed as median (interquartile range, IQR). Categorical variables were reported as n (%).

To balance the baseline characteristics of enrolled patients, propensity score matching (PSM) was performed. Propensity scores were estimated using a logistic regression model based on age, baseline BW, BMI, diabetes duration, HbA1c, eGFR, and insulin use at baseline. The PEG-Loxe group and SEMA group were matched at a 1:1 ratio using nearest neighbor matching with a caliper value of 0.02. After matching, intergroup balance was verified by standardized mean difference (SMD), with a threshold of SMD < 0.2 indicating acceptable balance. Differences in baseline characteristics between patients administrated with PEG-Loxe and s.c. semaglutide were compared before and after matching, using independent samples *t*-test, Mann-Whitney *U* test, or χ^2^ test.

Effectiveness analyses used a linear mixed model (LMM) with repeated measures that considered the least-squares mean (LSM) change from baseline to each postbaseline time point through 2 years as the response variable. We included treatment group, follow-up time, group-time interaction (to test for group-specific trends in efficacy over time) as fixed effects, and baseline HbA1c as a covariate. A patient-level random intercept was included to account for within-patient correlation of repeated measurements, with an unstructured covariance matrix for the error term. Between-group differences at specific time points were presented as the LSM difference with corresponding 95% confidence intervals (95%CI). Hypoglycemic and gastrointestinal adverse events were dichotomized as yes/no. The proportion of patients with at least one event per time period was calculated. Changes in categorical study endpoints were evaluated using χ^2^ test or Fisher exact for trend. Subgroup analyses based on the post-PSM full analysis set were performed using the primary analysis LMM. Interaction terms between subgroup factors and treatment groups were introduced to test whether treatment effects varied across subgroups.

Given the low proportion of missing data, missing data were excluded, and a sensitivity analysis for the primary study endpoint was conducted after re-performing PSM.

All statistical analysis was conducted using SPSS 26.0 for Windows (IBM SPSS Statistics, IBM Corporation, Armonk, NY). Graph-Pad Prism 8.2.0 (GraphPad Software Inc., San Diego, CA) was employed to perform figure generation. *P* values of < 0.05 (two tailed) were considered to indicate statistical significance.

## Results

**Figure 1:**
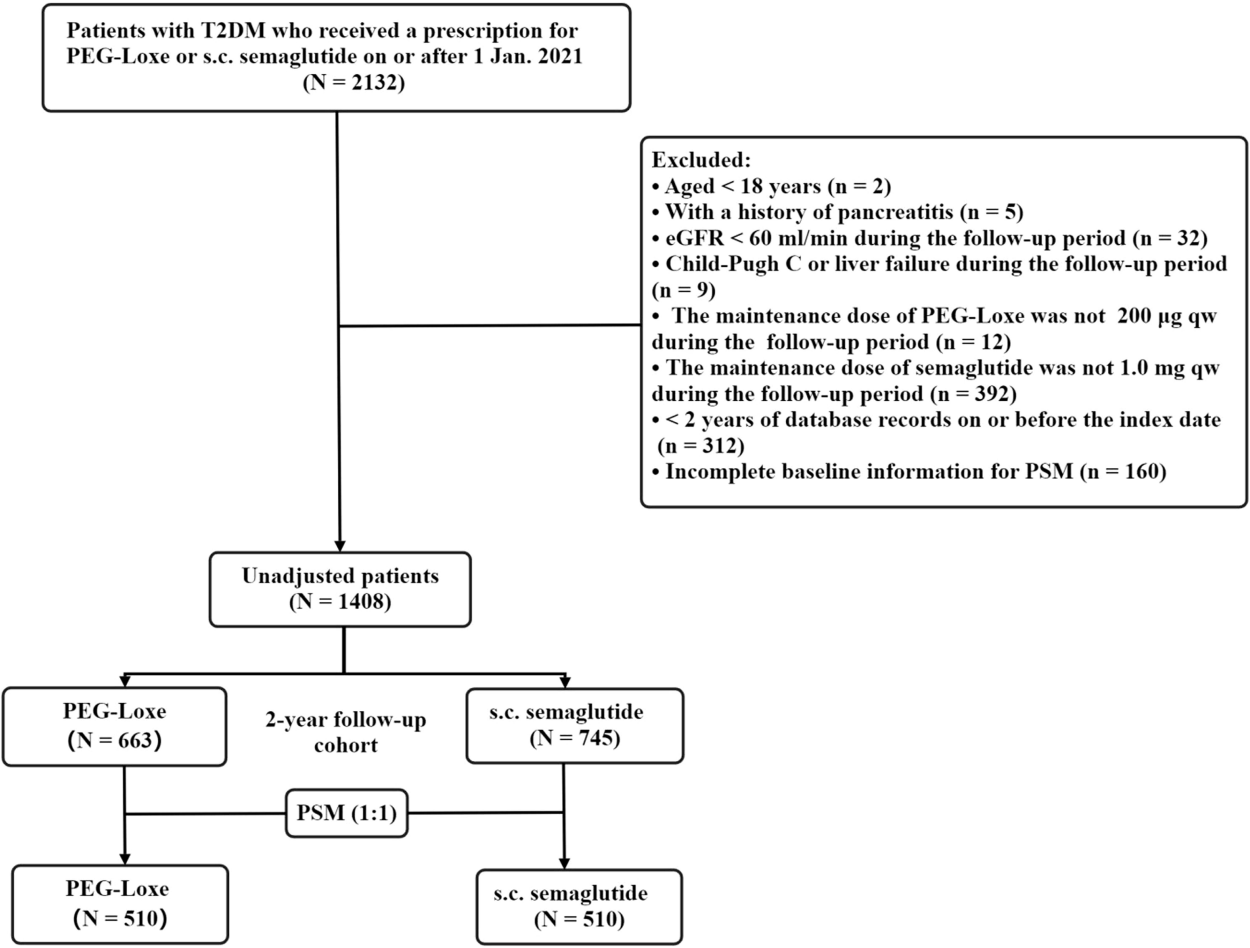
Flowchart diagram of the study. T2DM, type 2 diabetes mellitus; PEG-Loxe, polyethylene glycol loxenatide; s.c., subcutaneous; eGFR, estimated glomerular filtration rate; PSM, propensity score matching.

**Figure 1** depicted the study flowchart. From 2132 T2DM patients newly initiated on PEG-Loxe or s.c. semaglutide, 1408 (PEG-Loxe: 663; semaglutide: 745) met inclusion criteria. After 1:1 PSM, 510 patient pairs were analyzed.

Baseline demographics were shown in **Table 1**. In the original cohort, PEG-Loxe patients had longer T2DM duration, older age, lower BMI, LDL-C, Scr, eGFR, and higher ALT compared to semaglutide recipients. PEG-Loxe users more frequently used biguanides, α-glucosidase inhibitors, SGLT2 inhibitors, and long-acting insulin (**Supplementary Table 1**). PSM successfully balanced all baseline variables between groups (all *p* > 0.05). All baseline variables were balanced (all SMD < 0.2). The final matched cohort (mean age: 55.23 ± 15.22 years; 56.3% female) had a median follow-up of 2.0 years, with baseline HbA1c 9.20 ± 2.33%, BMI 25.91 ± 3.48 kg/m², and 41.46% on insulin.

**Table 1:** Baseline characteristics.

Baseline variables	Original data			Data after matching		
	PEG-Loxe (n = 663)	S.C. semaglutide (n = 745)	*P* value	PEG-Loxe (n = 510)	Semaglutide (n = 510)	*P* value
Age, years	58.00 (48.00, 67.00)	56.00 (40.00, 66.00)	0.001	56.00 (46.75, 66.00)	59.00 (42.00, 68.00)	0.385
Sex, female	363 (54.8)	402 (54.0)	0.766	288 (56.5)	286 (56.1)	0.900
BW, kg	66.00 (60.60, 74.00)	70.00 (62.00, 78.00)	<0.001	68.00 (63.00, 74.00)	67.00 (62.75, 74.00)	0.359
BMI, kg/m^2^	25.34 (23.03, 27.12)	26.03 (24.03, 27.68)	<0.001	25.64 (23.88, 27.43)	25.56 (23.88, 27.35)	0.891
Diabetes duration, years	7.00 (4.00, 11.00)	5.00 (2.00, 10.00)	<0.001	7.00 (4.00, 10.00)	6.00 (3.00, 11.00)	0.113
Baseline values of laboratory tests						
HbAlc, %	10.24 (8.76, 12.00)	10.02 (8.66, 12.10)	0.386	9.00 (7.48, 10.67)	8.73 (7.48, 10.90)	0.616
SBP, mmHg	132.00 (125.00, 139.00)	132.00 (125.00, 139.00)	0.891	132.00 (125.00, 136.00)	131.00 (124.00, 139.00)	0.596
DBP, mmHg	81.00 (76.00, 86.00)	80.00 (75.00, 89.00)	0.640	80.00 (76.00, 86.00)	81.00 (75.75, 89.25)	0.089
TC, mmol/L	5.10 (4.02, 6.03)	5.18 (4.38, 6.04)	0.194	5.01 (4.00, 5.92)	5.11 (4.28, 6.07)	0.097
TG, mmol/L	1.87 (1.27, 2.90)	1.83 (1.32, 2.74)	0.457	1.76 (1.23, 2.66)	1.68 (1.17, 2.68)	0.140
HDL-C, mmol/L	1.17 (0.96, 1.42)	1.21 (1.05, 1.41)	0.081	1.11 (0.94, 1.35)	1.17 (1.00, 1.36)	0.130
LDL-C, mmol/L	3.31 (2.74, 4.00)	3.61 (3.06, 4.28)	<0.001	3.32 (2.67, 3.95)	3.28 (2.69, 3.94)	0.963
ALT, U/L	25.47 (15.90, 37.61)	23.37 (17.06, 30.85)	0.008	24.68 (15.48, 37.31)	23.58 (17.24, 31.09)	0.163
AST, U/L	22.15 (15.74, 30.01)	20.85 (16.18, 25.19)	<0.001	21.59 (15.80, 29.99)	21.86 (16.95, 25.67)	0.077
Scr, μmol/L	82.44 (69.59, 98.10)	84.90 (71.67, 100.98)	0.004	82.58 (69.59, 98.42)	85.54 (71.03, 100.90)	0.059
eGFR mL/(min·1.73m^2^)	80.53 (60.71, 99.18)	84.10 (68.75, 100.81)	0.003	82.39 (62.40, 99.74)	83.69 (66.90, 108.32)	0.163
HGB, g/L	135.98 ± 20.67	135.14 ± 20.44	0.447	137.00 (122.75, 150.00)	137.00 (122.75, 147.25)	0.605
CVD history, yes						
Hypertension	240 (36.2)	310 (41.6)	0.038	173 (33.9)	175 (34.3)	0.895
Myocardial infarction	42 (6.3)	56 (7.5)	0.384	31 (6.1)	40 (7.8)	0.268
Heart failure	22 (3.3)	29 (3.9)	0.565	19 (3.7)	23 (4.5)	0.528
Ischemic stroke	53 (8.0)	62 (8.3)	0.822	38 (7.5)	46 (9.0)	0.362
CCI score	1.00 (0.00, 2.00)	1.00 (0.00, 2.00)	0.002	2.00 (0.00, 2.00)	1.00 (0.00, 2.00)	0.088
Diabetes Complications, yes						
Retinopathy	77 (11.6)	74 (9.9)	0.309	56 (11.0)	55 (10.8)	0.920
Neuropathy	115 (17.3)	122 (16.4)	0.627	84 (16.5)	88 (17.3)	0.738
Diabetic foot	20 (3.0)	15 (2.0)	0.228	16 (3.1)	13 (2.5)	0.572
Vasculopathy	110 (16.6)	119 (16.0)	0.754	74 (14.5)	80 (15.7)	0.600
aDCSI score	1.00 (0.00, 2.00)	0.00 (0.00, 2.00)	<0.001	1.00 (0.00, 1.00)	0.00 (0.00, 2.00)	0.866

Normally distributed continuous variables presented as mean (± SD) and non-normally distributed continuous variables presented as median (IQR). Categorical variables presented as n (%).

T2DM, type 2 diabetes mellitus; PEG-Loxe, polyethylene glycol loxenatide; s.c., subcutaneous; BW, body weight; BMI, body mass index; HbAlc, glycated hemoglobin; SBP, systolic pressure; DBP, diastolic blood pressure; TC, total cholesterol; TG, triglyceride; HDL-C, high-density lipoprotein cholesterol; LDL-C, low-density lipoprotein cholesterol; ALT, glutamic-pyruvic transaminase; AST, glutamic oxalacetic transaminase; Scr, serum creatinine; eGFR, estimated glomerular filtration rate; HGB, hemoglobin; CVD, cardiovascular disease; CCI, Charlson Comorbidity Index; aDCSI, adapted Diabetes Complication Severity Index.

Both groups achieved HbA1c reductions at month 3, sustained through month 24 (**Table 2**). LMM revealed a significant interaction effect between treatment groups and time on changes in HbA1c (*p* < 0.001), indicating that the s.c. semaglutide group exhibited a superior downward trend in HbA1c compared with the PEG-Loxe group. However, after 24 months, HbA1c decreased by LSM 2.02% (95% CI: -2.23, -1.81) with PEG-Loxe vs. LSM 2.23% (95% CI: -2.46, -2.00) with s.c. semaglutide. The estimated treatment difference (ETD) was -0.21% (95% CI -0.52, 0.10), a non-statistically significant variation (*p* = 0.260). Sensitivity analyses of the primary endpoints demonstrated similar results (**Supplementary Table 2**). Furthermore, all composite endpoints did not differ between groups (all *p* > 0.05, **Table 3**).

**Table 2:** Efficacy outcomes of PEG-Loxe versus s.c. semaglutide in the matched population^*^.

Endpoints^**^	Least-squares mean change (95%CI)		Estimated treatment difference (95% CI)	*P* value
	PEG-Loxe (n = 510)	S.C. semaglutide (n = 510)		
HbAlc, %				
T3	-0.58 (-0.81, -0.35)	-0.96 (-1.21, -0.71)	-0.38 (-0.72, -0.05)	0.061
T6	-1.23 (-1.44, -1.01)	-1.34 (-1.57, -1.11)	-0.12 (-0.43, 0.19)	0.514
T12	-1.99 (-2.20, -1.78)	-2.18 (-2.40, -1.95)	-0.19 (-0.49, 0.12)	0.259
T24	-2.02 (-2.23, -1.81)	-2.23 (-2.46, -2.00)	-0.21 (-0.52, 0.10)	0.260
BW, kg				
T3	-1.03 (-1.75, -0.31)	-3.05 (-4.31, -1.79)	-2.02 (-3.45, -0.58)	0.001
T6	-1.39 (-2.13, -0.65)	-4.22 (-5.48, -2.96)	-2.83 (-4.27, -1.39)	<0.001
T12	-1.30 (-2.15, -0.44)	-5.16 (-6.54, -3.79)	-3.87 (-5.46, -2.28)	<0.001
T24	-2.72 (-3.53, -1.92)	-6.27 (-7.74, -4.80)	-3.55 (-5.22, -1.88)	<0.001
BMI, kg/m^2^				
T3	-0.17 (-0.36, 0.02)	-0.82 (-1.24, -0.40)	-0.65 (-1.11, -0.19)	0.049
T6	-0.27 (-0.46, -0.08)	-1.03 (-1.46, -0.60)	-0.76 (-1.24, -0.29)	0.015
T12	-0.36 (-0.59, -0.12)	-0.98 (-1.41, -0.55)	-0.62 (-1.10, -0.15)	0.017
T24	-0.75 (-0.98, -0.51)	-1.57 (-2.00, -1.14)	-0.82 (-1.33, -0.32)	0.001
LDL-C, mmol/L				
T3	-0.35 (-0.47, -0.23)	-0.30 (-0.42, -0.18)	0.05 (-0.13, 0.23)	0.550
T6	-0.33 (-0.44, -0.23)	-0.54 (-0.66, -0.42)	-0.21 (-0.37, -0.05)	0.009
T12	-0.34 (-0.45, -0.24)	-0.86 (-0.99, -0.74)	-0.52 (-0.69, -0.35)	<0.001
T24	-0.35 (-0.46, -0.24)	-0.86 (-0.98, -0.75)	-0.51 (-0.68, -0.34)	<0.001
SBP, mmHg				
T3	-0.10 (-0.61, -0.04)	-1.11 (-2.94, -1.01)	-1.01 (-3.20, -0.14)	<0.001
T6	-0.19 (-0.91, -0.07)	-1.88 (-3.70, -0.62)	-1.69 (-5.07, -0.22)	<0.001
T12	-0.17 (-0.82, -0.06)	-2.01 (-3.70, -0.62)	-1.84 (-7.12, -0.14)	<0.001
T24	-0.18 (-0.82, -0.06)	-2.46 (-5.01, -0.43)	-2.08 (-9.33, -0.11)	<0.001
eGFR, mL/(min·1.73m^2^)				
T3	0.23 (-2.11, 2.58)	0.53 (-2.18, 3.22)	0.29 (-3.21, 3.80)	<0.001
T6	0.74 (-1.54, 3.02)	1.09 (-1.64, 3.83)	0.36 (-3.23, 3.94)	<0.001
T12	0.86 (-1.38, 3.10)	1.45 (-1.25, 4.16)	0.59 (-2.89, 4.07)	<0.001
T24	0.91 (-1.35, 3.16)	1.65 (-0.82, 4.13)	0.75 (-2.53, 4.03)	<0.001

^*^Least-squares means are from the statistical model described in the statistical analysis plan.

^**^Results are expressed as least-squares mean changes 3 (T3), 6 (T6), 12 (T12) and 24 (T24) months from baseline with their 95% confidence interval (95% CI).

PEG-Loxe, polyethylene glycol loxenatide; s.c., subcutaneous; HbAlc, glycated hemoglobin; BW, body weight; BMI, body mass index; LDL-C, low-density lipoprotein cholesterol; SBP, systolic blood pressure; eGFR, estimated glomerular filtration rate.

**Table 3:** Composite endpoints in the matched population.

Composite endpoints^*^ n (%)	Visit	PEG-Loxe (n = 510)	S.C. semaglutide (n = 510)	Estimated odds ratio^**^ (95% CI)	*P* value
HbA1c < 7.0% without hypoglycemia and no BW gain	T3	86 (16.9)	109 (21.4)	0.746 (0.545 - 1.021)	0.068
	T6	111 (21.8)	134 (26.3)	0.773 (0.579 - 1.031)	0.079
	T12	178 (34.9)	187 (36.7)	0.926 (0.717 - 1.196)	0.557
	T24	209 (41.0)	231 (45.3)	0.839 (0.654 - 1.075)	0.164
HbA1c ≤ 6.5% without hypoglycemia and no BW gain	T3	58 (11.4)	74 (14.5)	0.756 (0.523 - 1.092)	0.136
	T6	83 (16.3)	102 (20.0)	0.778 (0.565 - 1.071)	0.123
	T12	100 (19.6)	122 (23.9)	0.776 (0.575 - 1.046)	0.095
	T24	124 (24.3)	146 (28.6)	0.801 (0.606 - 1.059)	0.119
HbA1c reduction ≥1% without hypoglycemia and no BW gain	T3	196 (38.4)	220 (43.1)	0.823 (0.641 - 1.057)	0.126
	T6	227 (44.5)	234 (45.9)	0.946 (0.739 - 1.211)	0.660
	T12	257 (50.4)	268 (52.5)	0.917 (0.717 - 1.173)	0.491
	T24	274 (53.7)	282 (55.3)	0.939 (0.734 - 1.201)	0.615

^*^Composite endpoints were analyzed at 3 (T3), 6 (T6), 12 (T12) and 24 (T24) months following the baseline.

^**^The odds ratios and associated CIs are estimated from a logistic regression model.

PEG-Loxe, polyethylene glycol loxenatide; s.c., subcutaneous; HbAlc, glycated hemoglobin; BW, body weight.

Semaglutide demonstrated superior weight loss through follow-up period. The ETD at 24 months was -3.55 kg (95% CI: -5.22, -1.88) (*p* < 0.001, **Table 2**). BMI reductions were also significantly greater with semaglutide over the course of follow-up except at month 3 (*p* = 0.049). No statistically significant difference was observed; however, the confidence intervals do not exclude potentially meaningful clinical differences. At month 24, the ETD was -0.82 kg/m² (95% CI: -1.33, -0.32; *p* = 0.001).

Semaglutide provided greater reductions in LDL-C at month 6, with sustained benefits through month 24. The ETD at 24 months was -0.51 mmol/L (95% CI: -0.68, -0.34; *p* < 0.001). SBP reductions was significantly greater with semaglutide at all visits (all *p* < 0.001), with a maximum difference of -2.08 mmHg (95% CI -9.33, -0.11). Renal function improvements favored semaglutide during the follow-up period (all *p* < 0.001, ETD at 24 months: 0.75 mL/(min·1.73m^2^), 95% CI -2.53, 4.03).

No significant changes in concomitant antihyperglycemic, antihypertensive, and lipid-lowering treatment regimen were observed (**Supplementary Table 3**). Over the course of follow-up, medication adherence levels exhibited no statistically significant difference between the two groups (**Supplementary Table 4**).

Severe hypoglycemia occurred in 1 (0.2%) of patients in both groups. Gastrointestinal adverse events (nausea, vomiting, diarrhea) were more frequent with s.c. semaglutide during the first 3 months (*p* < 0.05, **Table 4**).

**Table 4:** Adverse drug events in the matched population.

ADEs, n (%)^*^	Time interval	PEG-Loxe (n = 510)	S.C. semaglutide (n = 510)	*P* value
Hypoglycemia				
Level 1	0~3 months	11 (2.2)	14 (2.7)	0.544
	3~6 months	10 (2.0)	9 (1.8)	0.817
	6~12 months	3 (0.6)	5 (1.0)	0.478
	12~24 months	0 (0.0)	2 (0.4)	0.157
Level 2	0~3 months	6 (1.2)	8 (1.6)	0.590
	3~6 months	4 (0.8)	5 (1.0)	0.738
	6~12 months	0 (0.0)	0 (0.0)	–
	12~24 months	0 (0.0)	0 (0.0)	–
Level 3	0~3 months	1 (0.2)	1 (0.2)	
	3~6 months	0 (0.0)	0 (0.0)	–
	6~12 months	0 (0.0)	0 (0.0)	–
	12~24 months	0 (0.0)	0 (0.0)	–
Gastrointestinal disorder				
Nausea	0~3 months	18 (3.5)	60 (11.8)	<0.001
	3~6 months	6 (1.2)	14 (2.7)	0.071
	6~12 months	0 (0.0)	1 (0.2)	0.317
	12~24 months	0 (0.0)	0 (0.0)	–
Vomiting	0~3 months	18 (3.5)	42 (8.2)	0.001
	3~6 months	3 (0.6)	8 (1.6)	0.130
	6~12 months	0 (0.0)	0 (0.0)	–
	12~24 months	0 (0.0)	0 (0.0)	–
Diarrhea	0~3 months	12 (2.4)	26 (5.1)	0.021
	3~6 months	2 (0.4)	5 (1.0)	0.255
	6~12 months	0 (0.0)	0 (0.0)	–
	12~24 months	0 (0.0)	0 (0.0)	–

^*^n (%), Number and percentage of patients experiencing at least one event.

Level 1, Blood glucose 3.0-3.9 mmol/L; Level 2, Blood glucose < 3.0 mmol/L; Level 3, Severe hypoglycemia requiring assistance.

ADEs, adverse drug events.

**Figure 2:**
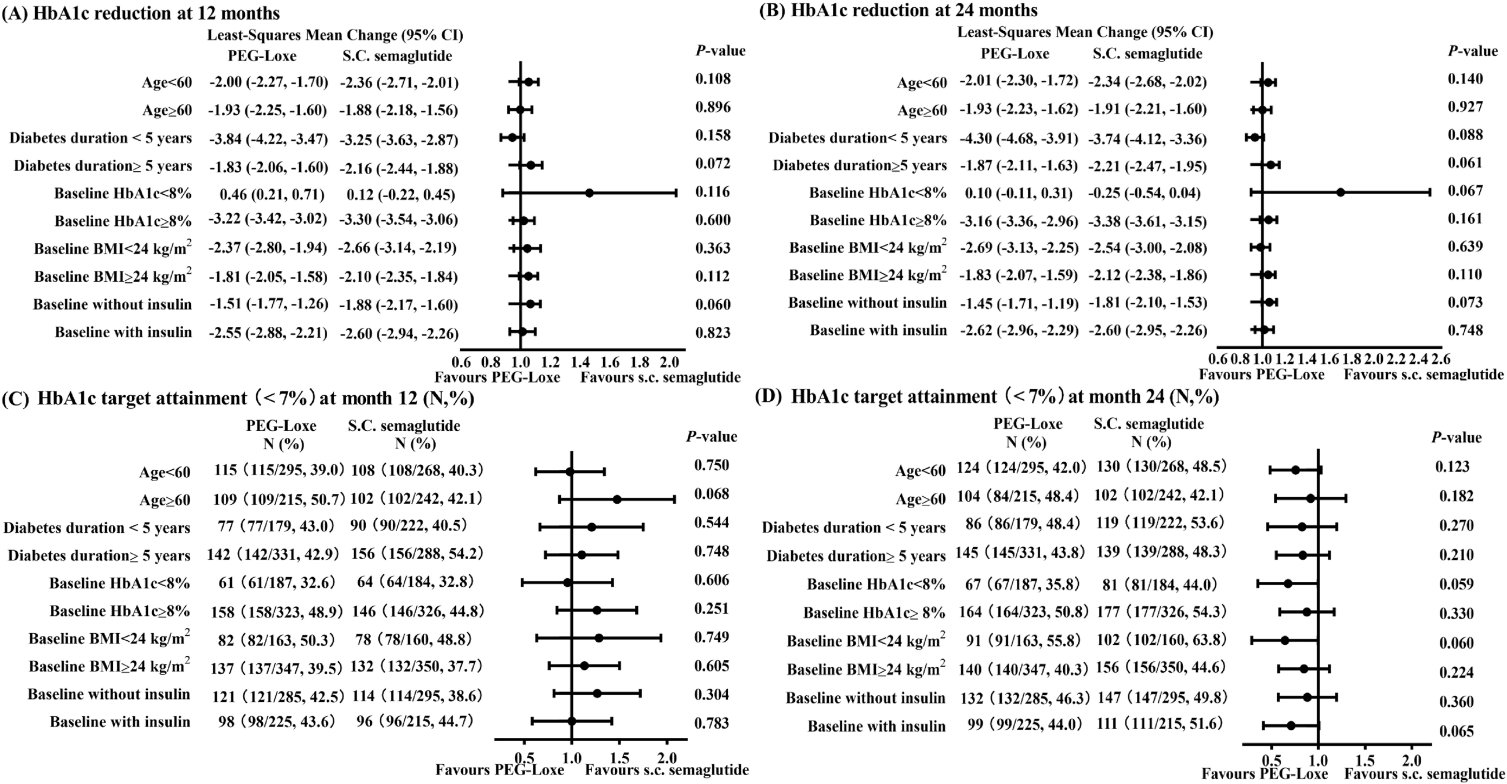
HbA1c least-squares mean changes or target attainment across subgroups. **(A)** HbA1c reduction at month 12. **(B)** HbA1c reduction at month 24. **(C)** Proportion of patients achieving HbA1c < 7.0% at month 12 (N, %). **(D)** Proportion of patients achieving HbA1c < 7.0% at month 24 (N, %). HbAlc, glycated hemoglobin; BW, body weight; PEG-Loxe, polyethylene glycol loxenatide; BMI, body mass index.

No significant interaction effect in HbA1c changes (all *p* > 0.05), or notable differences in HbA1c target attainment (%) (all *p* > 0.05) were observed across subgroups stratified by age, diabetes duration, baseline HbA1c, BMI, or insulin use (**Figure 2**).

## Discussion

To the best of our knowledge, this represents the first real-world, head-to-head comparison evaluating the glycemic control, exploratory cardiorenal indicators and safety of once-weekly PEG-Loxe (200 μg) versus s.c. semaglutide (1.0 mg) in Chinese patients with T2DM over a 24-month period. After adjusting for baseline confounding factors (a limitation unavoidable in retrospective studies) with PSM, compared with s.c. semaglutide, receiving PEG-Loxe resulted in comparable HbA1c reductions and target attainment rates, sustaining after 24 months. Greater improvements in BW and cardiorenal parameters (LDL-C, BP, and eGFR) were observed in s.c. semaglutide group. PEG-Loxe appeared to demonstrate better gastrointestinal tolerability during the initial treatment period.

Sustained decreases in HbA1c were observed in both groups throughout the 24-month period, with non-significant ETD, further confirming the real-world glucose-lowering efficacy of PEG-Loxe in the Chinese population. PEG-Loxe 200 μg treatment resulted in the LSM change from baseline in HbA1c of -1.23% after 6 months in this study, which is comparable to previous RCTs (1.14%~1.34%) (7, 8). On the other hand, HbA1c decreased by -2.18% after a 12-month s.c. semaglutide treatment, different with that reported in a previous real-world study (-0.96%) (20), which may due to the possible positive relationship between baseline HbA1c (9.17 ± 2.36% vs. 7.8 ± 1.3%) and margins of reduction of HbA1c. However, two meta-analyses published recently (16, 21) consistently showed that s.c. semaglutide 1.0 mg resulted in a greater reduction in HbA1c than weekly PEG-Loxe 200 μg (range of ETD in reduction: -0.36% to -0.45%). The ETD of HbA1c reduction observed in our study (-0.20% after 24 months of follow-up) was less pronounced than that reported in the aforementioned meta-analyses. This difference may be mainly attributed to study design: real-world studies recruited patients with more complicated disease status and higher baseline blood glucose levels, followed for as long as 24 months; by contrast, the RCTs included in meta-analyses featured relatively short follow-up periods (12–40 weeks) and strict patient selection criteria.

Sustaining superior BW and BMI reductions were observed with s.c. semaglutide than those with PEG-Loxe over a 24-month period. The ETD in weight after 12 months of treatment was -3.87 kg (**Table 2**), which was in line with the findings from prior meta-analyses (16, 21) (range of ETD at 12 months: -2.63 kg to -5.16 kg). The potential reason lies in the distinct central effects induced by the two drugs in modulating appetite. Semaglutide’s inhibitory action on central appetite is mainly mediated by activating GLP-1 receptors located in the hypothalamus and medulla oblongata (22). PEG-Loxe, a large albumin-based molecule (relative molecular mass 44 kDa), may hardly cross blood-brain barrier (BBB) or diffuse into the brain at the area postrema (vomiting center) or hypothalamus (center for the regulation of appetite and food ingestion) (23).

Semaglutide led to significantly greater improvements in LDL-C than with PEG-Loxe in our clinical observation, which was consistent with the findings of previous meta-analyses (16, 21) and might be also partly due to the different BW/BMI benefits documented here. As identified in the SELECT trial, semaglutide could improve cardiovascular outcomes by reducing metabolically dysfunctional body fat (24). Given the difference in baseline BMI between patients enrolled in the present study and the SELECT trial (25.56 kg/m² vs. ≥ 27 kg/m²), the cardiovascular outcomes of patients treated with semaglutide in this study required further exploration. Other mechanisms by which semaglutide improves lipid profiles may involve decreasing ApoB-48 synthesis, augmenting ApoB-48 catabolism, and exerting anti-inflammatory effects (25, 26).

PEG-Loxe lagged behind semaglutide in lowering SBP, which was in agreement with the outcomes of the earlier meta-analysis (16, 21). Semaglutide’s superiority in reducing SBP may be in part related to its marked BW reduction effect (27). Additional mechanisms of improving SBP may involve enhancing endothelial function, modulating smooth muscle cell phenotype, activating GLP-1 receptors located in the brainstem, and mediating natriuretic actions (28, 29).

Few published studies have compared PEG-Loxe with s.c. semaglutide regarding their renal effects. Semaglutide provided greater improvement in eGFR than PEG-Loxe in this study, possibly due to its direct effectiveness on renal tissue involving reducing inflammation, oxidative stress, and fibrosis, confirmed in FLOW study (2). The FLOW study showed pronounced beneficial effects of semaglutide on renal function in comparison with the placebo group [the mean annual eGFR slope was less steep by 1.16 mL/(min·1.73m^2^)]. However, in the present study, the magnitude of change [ETD: 0.75 mL/(min·1.73m^2^)] might fall within the range of biological and measurement variability. The clinical implication of this difference seemed limited, warranting further evaluation.

Throughout the 24-month follow-up duration of this research, no notable difference was observed in the rate of hypoglycemic episodes between the two groups, corroborating the indirect comparison outcomes reported in previous meta-analyses (16, 21). Predominantly, GLP-1RAs lower blood glucose levels through the stimulation of glucose-dependent insulin secretion and are unlikely to trigger hypoglycemia. However, the co-administration of insulin/oral sulfonylurea medications may result in a heightened likelihood of hypoglycemia occurrence (30).

As expected, gastrointestinal AEs were the most common adverse events in our study. The pro- portions of patients reporting gastrointestinal disorder were higher for s.c. semaglutide than PEG-Loxe within the initial 3 months. Statistical results from previous meta-analyses (16, 21) suggested that PEG-Loxe was linked to a higher incidence of gastrointestinal disturbances relative to semaglutide, yet the risk of GLP-1RA discontinuation attributable to semaglutide-related gastrointestinal side effects was higher than that with PEG-Loxe. In general, gastrointestinal manifestations associated with semaglutide occur predominantly within weeks 8–12 post-dose escalation and resolve progressively over the course of treatment (31). Taking into account the greater severity of semaglutide-induced gastrointestinal discomfort-which contributes to a heightened risk of treatment withdrawal-this may underlie the higher count of early-stage semaglutide-related gastrointestinal events documented in the EMRs of the current research.

HbA1c reduction with GLP-1RAs may be mainly influenced by baseline HbA1c value and individual age (32). In general, the efficacy of GLP-1RAs on glycemic control is based on baseline level, without uniform margin of reduction, thus avoiding frequent occurrences of hypoglycemia (33). Our analysis revealed similar HbA1c reductions between the treatment groups among various baseline HbA1cs subgroups over time, contributing to a further confirming of aforementioned alike efficacy of glycemic control with PEG-Loxe compared with s.c. semaglutide. On the other hand, similar HbA1c variations between treatments across age subgroups were observed at each visit, indicating that the age-related decrease of β-cell sensitivity to incretin (34) also did not influence the similar hypoglycemic effect between the two groups.

This real-world study has several limitations. Our cohort included Chinese single-ethnic T2DM population. Additionally, despite the use of PSM, residual confounding remains likely. Variables such as dietary patterns, lifestyle behaviors, socioeconomic status, and physician prescribing preferences were not available in the EMRs and therefore could not be adjusted for. Moreover, MARS 5-based adherence assessment might lack real-world accuracy. Inconsistent or missing records could cause bias. Finally, passive collection of data from EMRs might result in underestimating ADEs, especially non- serious gastrointestinal symptoms commonly associated with GLP-1RAs.

In summary, our real-life study showed that both at approved high dose, PEG-Loxe was as effective as s.c. semaglutide in improving HbA1c in T2DM patients, with likely less gastrointestinal disorder occurrence during the initial period of treatment. Subcutaneous semaglutide provided superior benefits in BW loss, lipid profile improvement, SBP reduction, and enhancing eGFR levels. In light of these findings, the importance of identifying subjects who would benefit the most from the application of PEG-Loxe, specifically, individuals with inadequately controlled glycemia who do not have chronic kidney disease or cardiovascular complications, is highlighted. For patients with overweight/obesity or established cardiorenal metabolic risk factors, semaglutide may confer greater additional benefits. These findings offer evidence to further assist in clinical decision making and care in this patient population. A well-designed, larger sample size with longer follow-up research may enable better validating the perspective of the present study.

## Data Availability

The original contributions presented in the study are included in the article/**Supplementary Material**. Further inquiries can be directed to the corresponding authors.
