# Supplementary material for: Real-world glycemic control, exploratory cardiorenal indicators, and safety of polyethylene glycol loxenatide versus semaglutide in type 2 diabetes patients: a Chinese two-center retrospective cohort study
Source: Front Endocrinol (Lausanne). 2026 Mar 24;17:1756581. doi: 10.3389/fendo.2026.1756581 (PMC13053266; doi:10.3389/fendo.2026.1756581)
Supplement: Supplementary file 1 [file DataSheet1.docx]

Supplementary Material

Graphical abstract


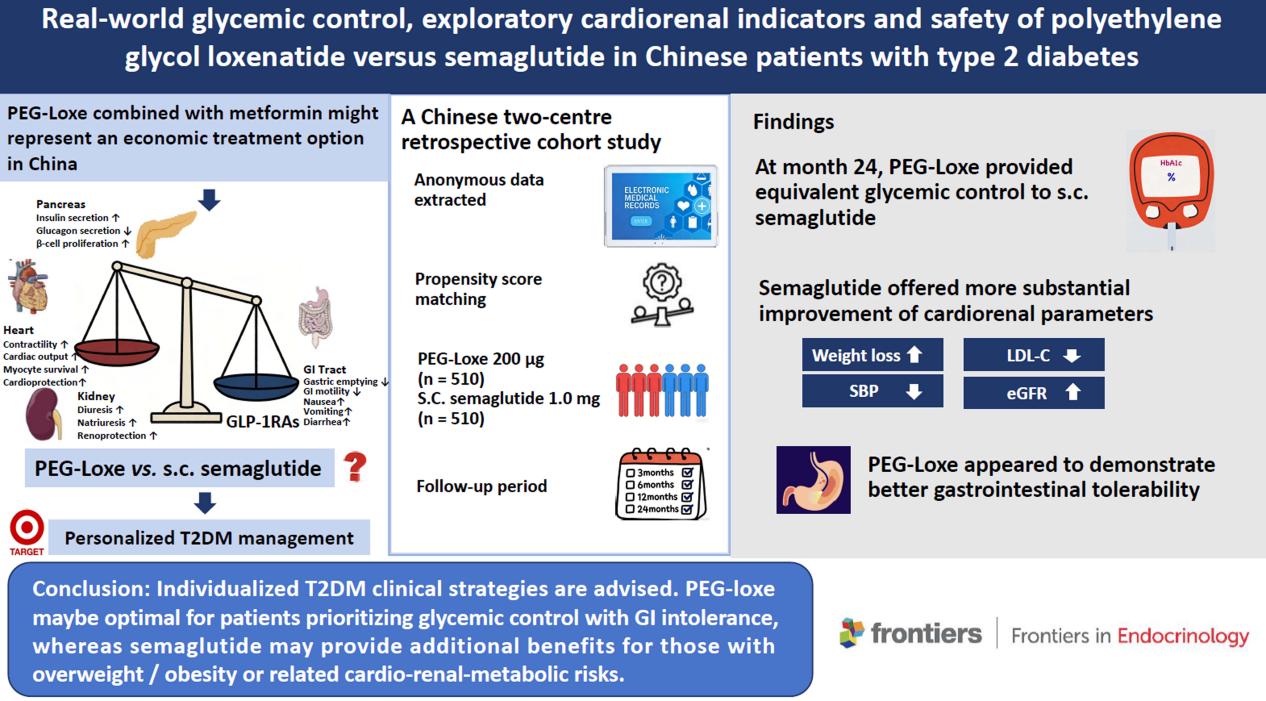


TABLE S1 Baseline detailed medication histories

|  | Original data | | | Data after matching | | |
| --- | --- | --- | --- | --- | --- | --- |
| Baseline antihyperglycemic agents, yes | PEG-Loxe  (n = 663) | S.C. semaglutide  (n = 745) | *P* value | PEG-Loxe  (n = 510) | Semaglutide  (n = 510) | *P* value |
| Biguanides | 217 (32.7) | 305 (40.9) | 0.001 | 181 (35.5) | 175 (34.3) | 0.693 |
| Sulphonylureas | 103 (15.5) | 105 (14.1) | 0.447 | 80 (15.7) | 81 (15.9) | 0.931 |
| Thiazolidinediones | 163 (24.6) | 187 (25.1) | 0.823 | 130 (25.5) | 133 (26.1) | 0.830 |
| α-Glycosidase Inhibitors | 97 (14.6) | 57 (7.7) | <0.001 | 54 (10.6) | 54 (10.6) | 1.000 |
| SGLT2i | 358 (54.0) | 337 (45.2) | 0.001 | 246 (48.2) | 241 (47.3) | 0.754 |
| Rapid-acting insulin | 48 (7.2) | 39 (5.2) | 0.119 | 26 ( 5.1) | 29 (5.7) | 0.677 |
| Long-acting insulin | 144 (21.7) | 129 (17.3) | 0.037 | 97 (19.0) | 91 (17.8) | 0.628 |
| Pre-mixed insulin | 152 (22.9) | 146 (19.6) | 0.127 | 102 (20.0) | 104 (20.4) | 0.876 |
| TDI^*^, IU | 0.00 (0.00, 28.00) | 0.00 (0.00, 22.00) | <0.001 | 0.00 (0.00, 24.00) | 0.00 (0.00, 24.50) | 0.774 |
| Lipid-lowering treatment, yes | 232 (35.0) | 242 (32.5) | 0.320 | 163 (32.0) | 169 (33.1) | 0.688 |

Non-normally distributed continuous variables presented as median (IQR). Categorical variables presented as n (%).

^*^TDI: Total daily insulin dose (calculated for patients on combined insulin therapy)

SGLT2i, sodium-glucose cotransporter-2 inhibitors

TABLE S2 Sensitivity analyses of the primary endpoint at 24 months

|  | Least-Squares Mean Change (95 %CI) | | Estimated treatment  difference (95% CI) | P value |
| --- | --- | --- | --- | --- |
|  | PEG-Loxe  (n = 488) | SEMA  (n = 488) |  |  |
| **HbA1c/ %** |  |  |  |  |
| T24 | -2.05 (-2.27, -1.83) | -2.26 (-2.50, -2.03) | -0.21 (-0.53, 0.11) | 0.239 |

TABLE S3 Concomitant antihyperglycemic, antihypertensive, and lipid-lowering treatment regimens in the matched population

| Visit | Treatment Category | Drug Class, yes, n (%) | PEG-Loxe  (n = 510) | S.C. semaglutide  (n = 510) | *P* value |
| --- | --- | --- | --- | --- | --- |
| T3 | Antihyperglycemic agents | Biguanides | 214 (42.0) | 190 (37.3) | 0.124 |
|  |  | Sulphonylureas | 64 (12.5) | 61 (12.0) | 0.775 |
|  |  | Thiazolidinediones | 133 (26.1) | 127 (24.9) | 0.666 |
|  |  | α-Glycosidase Inhibitors | 44 (8.6) | 43 (8.4) | 0.911 |
|  |  | SGLT2i | 256 (50.2) | 249 (48.8) | 0.661 |
|  |  | Rapid-acting insulins | 25 (4.9) | 28 (5.5) | 0.672 |
|  |  | Long-acting insulins | 83 (16.3) | 75 (14.7) | 0.489 |
|  |  | Pre-mixed insulins | 117 (22.9) | 115 (22.5) | 0.881 |
|  |  | TDI, IU, mean (SD) | 13.75 ± 19.70 | 13.91 ± 20.59 | 0.842 |
|  | Antihypertensive treatment | Total | 199 (39.0) | 200 (39.2) | 0.949 |
|  | Lipid-lowering treatment | Total | 188 (36.9) | 189 (37.1) | 0.948 |
| T6 | Antihyperglycemic agents | Biguanides | 216 (42.4) | 202 (39.6) | 0.373 |
|  |  | Sulphonylureas | 62 (12.2) | 60 (11.8) | 0.847 |
|  |  | Thiazolidinediones | 140 (27.5) | 123 (24.1) | 0.224 |
|  |  | α-Glycosidase Inhibitors | 44 (8.6) | 41 (8.0) | 0.734 |
|  |  | SGLT2i | 255 (50.0) | 261 (51.2) | 0.707 |
|  |  | Rapid-acting insulins | 23 (4.5) | 22 (4.3) | 0.879 |
|  |  | Long-acting insulins | 80 (15.7) | 70 (13.7) | 0.377 |
|  |  | Pre-mixed insulins | 112 (22.0) | 110 (21.6) | 0.879 |
|  |  | TDI, IU, mean (SD) | 13.68 ± 19.59 | 13.76 ± 20.37 | 0.802 |
|  | Antihypertensive treatment | Total | 194 (38.0) | 190 (37.3) | 0.796 |
|  | Lipid-lowering treatment | Total | 193 (37.8) | 192 (37.6) | 0.948 |
| T12 | Antihyperglycemic agents | Biguanides | 214 (42.0) | 200 (39.2) | 0.372 |
|  |  | Sulphonylureas | 62 (12.2) | 59 (11.6) | 0.771 |
|  |  | Thiazolidinediones | 139 (27.3) | 124 (24.3) | 0.283 |
|  |  | α-Glycosidase Inhibitors | 44 (8.6) | 40 (7.8) | 0.649 |
|  |  | SGLT2i | 264 (51.8) | 262 (51.4) | 0.900 |
|  |  | Rapid-acting insulins | 21 (4.1) | 22 (4.3) | 0.876 |
|  |  | Long-acting insulins | 79 (15.5) | 69 (13.5) | 0.374 |
|  |  | Pre-mixed insulins | 110 (21.6) | 108 (21.2) | 0.879 |
|  |  | TDI, IU, mean (SD) | 13.68 ± 19.58 | 13.60 ± 19.96 | 0.811 |
|  | Antihypertensive treatment | Total | 194 (38.0) | 189 (37.1) | 0.746 |
|  | Lipid-lowering treatment | Total | 195 (38.2) | 196 (38.4) | 0.949 |
| T24 | Antihyperglycemic agents | Biguanides | 215 (42.2) | 202 (39.6) | 0.408 |
|  |  | Sulphonylureas | 63 (12.4) | 59 (11.6) | 0.700 |
|  |  | Thiazolidinediones | 141(27.6) | 126 (24.7) | 0.285 |
|  |  | α-Glycosidase Inhibitors | 46 (9.0) | 41 (8.0) | 0.575 |
|  |  | SGLT2i | 268 (52.5) | 266 (52.2) | 0.900 |
|  |  | Rapid-acting insulins | 21 (4.1) | 19 (3.7) | 0.747 |
|  |  | Long-acting insulins | 78 (15.3) | 69 (13.5) | 0.422 |
|  |  | Pre-mixed insulins | 110 (21.6) | 107 (21.0) | 0.818 |
|  |  | TDI, IU, mean (SD) | 13.48 ± 19.18 | 13.41 ± 19.55 | 0.840 |
|  | Antihypertensive treatment | Total | 195 (38.2) | 189 (37.1) | 0.698 |
|  | Lipid-lowering treatment | Total | 199 (39.0) | 196 (38.4) | 0.847 |

TDI: Total daily insulin dose (calculated for patients on combined insulin therapy)

PEG-Loxe, polyethylene glycol loxenatide; S.C., subcutaneous; SGLT2i, sodium-glucose co-transporter-2 inhibit-

ors

TABLE S4 Medication adherence in the matched population

|  | Time interval | PEG-Loxe  (n = 510) | S.C. semaglutide  (n = 510) | P value |
| --- | --- | --- | --- | --- |
| Adherent^*^, n (%) |  |  |  |  |
|  | T0 | 389 (76.3) | 412 (80.8) | 0.079 |
|  | T12 | 378 (74.1) | 403 (79.0) | 0.065 |
|  | T24 | 375 (73.5) | 392 (76.9) | 0.218 |

^*^We defined adherent as a 5-item Medication Adherence Report Scale (MARS-5) score of 25 and non-adherent as a score of < 25 according to the literature.

T0: at the time of the patient’s first diagnosis of type 2 diabetes mellitus.

T12, T24: 12 and 24 months after receiving the first prescription of PEG-Loxe or s.c. semaglutide.
